# Long term follow up results of sequential left internal thoracic artery grafts on severe left anterior descending artery disease

**DOI:** 10.1186/1749-8090-5-87

**Published:** 2010-10-19

**Authors:** Murat Mert, Gurkan Cetin, Cenk Eray Yildiz, Murat Ugurlucan, Ilker Murat Caglar, Ahmet Ozkara, Atif Akcevin, Cihat Bakay

**Affiliations:** 1 Department of Cardiovascular Surgery, Instiute of Cardiology, Istanbul University, Istanbul, Turkey; 2 Department of Cardiovascular Surgery, Duzce Ataturk State Hospital, Duzce, Turkey; 3 Department of Cardiology, Duzce Ataturk State Hospital, Duzce, Turkey

## Abstract

**Purpose:**

Several alternative procedures have been proposed to achieve complete revascularization in the presence of diffuse left anterior descending coronary artery (LAD) disease. With the extensive use of internal thoracic artery grafts in coronary artery bypass procedures, sequential anastomosis of the left internal thoracic artery (LITA) to LAD has gained popularity in these challenging cases. The long term results of sequential LITA to LAD anstomosis were examined in this study.

**Patients and Methods:**

In order to determine the long term results of the sequential revascularization of LAD by LITA graft, 41 out of 49 patients operated between January 2001 and December 2005 were selected for control coronary arteriography. The median period for control coronary arteriography was 64 months.

**Results:**

Seventy five anastomoses were found to be fully patent (91,46%) among the 82 sequential LITA anastomoses (41 LITA grafts) on the LAD at a median follow-up period of 64 months (53 to 123 months). Among the 41 LITA grafts used for this purpose, 36 were found intact (complete patency of the proximal and distal anastomoses) (87,8%). Two LITA grafts (4 anastomoses) were found to be totally occluded (4,87%). The proximal anastomosis of the LITA graft was observed to be 90% stenotic in one patient (1,21%). In one patient tight stenosis of the distal anastomosis line was observed (1,21%), while in another patient 70% narrowing of LITA lumen after the proximal anastomosis was detected (1,21%).

**Conclusion:**

We strongly beleive that sequential LITA grafting of LAD is a safe alternative in the presence of severe LAD disease to achieve complete revascularization of the anterior myocardium with patency rates not much differing from conventional single LITA to LAD anastomosis.

## Introduction

The primary goal in coronary artery surgery is the complete revascularization with its proven superior long term results [1]. However, in some patients, the usual coronary bypass techniques may not allow a complete myocardial revascularization due to the extent of the disease. In such cases, complementary revascularization techniques may become mandatory especially if the diseased vessel is the LAD. In consequence, some alternative procedures, such as the use of multiple or sequential anastomoses [2], composite grafts [3], vein patch reconstruction [4] or coronary endarterectomy [1] have been proposed to revascularize the entire LAD system in the presence of diffuse disease.

Among the alternative procedures, sequential use of the left internal thoracic artery (LITA) is the preferred approach by our surgical team to overcome the diffuse LAD disease. The purpose of this study is to report the long term results of this procedure.

## Patients and Methods

In order to determine the long term results of the sequential revascularization of LAD by a LITA graft, 41 out of 49 patients, operated between January 2001 and December 2005, were selected for control coronary arteriography studies. Thirty one of the patients were male where as 10 were female. Age ranged between 44 and 72 (59,2 ± 7,0) years. Hypertension, diabetes mellitus, hyperlipidemia, chronic obstructive pulmonary disease and positive family history were present in 43%, 46%, 58%, %17 and 21% of the patients, respectively. Active or previous cigarette smoking history was present in 30 patients (73%). Pre-operative ejection fraction ranged between 35% and 51% (41,4 ± 4,5%). Regular anti-aggregant, lipid lowering or anti-ischemic medication usage was inhomogenious and could not be clearly identified; however, all the patients were prescribed either a calcium channel blocker or a beta-blocker, and aspirin and a statin agent after the surgery. Patients operated on emergent basis, operated on off-pump fashion, whom requiring additional cardiovascular procedures other than coronary revascularization, and who have chronic renal failure were excluded from the study.

In all patients, LITA was used to revascularize the LAD sequentially in order to by-pass proximal and mid portion stenoses in the artery. In addition to sequential LITA anastomoses, 109 anastomoses were performed with saphenous vein grafts (37 for the right coronray artery, 43 for the obtuse marginal branches of the circumflex coronary artery and 29 for the diagonal branch of the LAD). The demographic data of the patients are presented on Table 1. The median period for control coronary arteriography was 64 months (range 53 to 123 months).

**Table 1:** Demographic Data of the Study Group

Age (years): 59,2 ± 7,0 (range 44 to 72)
Male/Female: 31/10

Hypertension: 18/41 (43%)

Diabetes Mellitus: 19/41 (46%)

COPD: 7/41 (17%)

Hyperlipidemia: 24/41 (58%)

Family History: 9/41 (21%)

Cigarette Smoking: 30/41 (73%)

Pre-op EF: 41,4 ± 4,5% (range 35% to 51%)

(COPD, Chronic Obstructive Pulmonary Disease; Pre-op, Pre-operative; EF, Ejection fraction)

### Surgical technique

The sternum was opened via sternotomy incision. The LITA was harvested with a large pedicle containing both veins by the aid of electrocautery. Following systemic heparinization, the LITA was transected after its bifurcation and was kept in papaverine-soaked sponge until its use. The cardiopulmonary bypass was initiated with aortic and right atrial cannulations. Following a period of cooling to 28-32°C, the aorta was cross-clamped and cardioplegic arrest was established by cold blood cardioplegia infused through the aortic root and the coronary sinus which was repeated every 20 minutes. First, the saphenous vein distal anastomoses were performed and followed by LAD arteriotomy between the estimated proximal and mid-stenosis of this artery. 1,5 mm and 1 mm coronary artery probes were introduced distally through this hole on the LAD and if the 1 mm probe could not be passed through the suspected mid LAD stenosis, another arteriotomy was performed on LAD distal to this stenosis region. Then, arteriotomy was made on mid portion of LITA and at this region the LITA was anastomosed side-to-side to the proximal LAD where as the LITA end was anastomosed in an end-to-side fashion to distal LAD sequentially bypassing the stenoses. Care was carried to prevent bleeding from LITA and from the distal LAD arteriotomy to check the patency of the proximal LAD anastomosis. The aortic clamp was then released and the proximal anastomoses were performed during the re-warming period under a partial aortic clamp. Following the warming period, the patient was weaned off the cardiopulmonary bypass and the chest was closed after completion of hemostasis.

### Control coronary arteriography

The coronary arteriographies were performed after explaining the aim in details and obtaining patient consent through the right femoral artery with Philips Integris H 3000 and Philips Integris HM 3000 C devices equipped with Quinton monitorization systems (Philips Company, Eindhoven, The Netherlands). All stenoses of LITA greater than 50% were defined as "graft stenosis", and the non-visualization of the contrast material after a certain point of the graft, at the anastomosis line or non-filling of the host coronary artery, was defined as "graft occlusion".

## Results

### Post-operative period

There was no operative mortality among the 49 patients operated during the study period. For the angiographically controlled 41 patients, the mean aortic cross-clamp time was 79 ± 21,43 minutes and the mean cardiopulmonary bypass time was 129,11 ± 33,23 minutes. The mean number of distal anastomoses performed per patient was 4,65 ± 0,62. One patient required intra-aortic balloon pump assistance to wean off the cardiopulmonary bypass (2,4%). Two patients (4,8%) were taken back to the operating theatre due to bleeding and hemostasis was performed. Perioperative myocardial infraction characterized by new Q wave appearance on the postoperative electrocardiography was diagnosed in one patient (2,4%) and was confined to the inferior border. Left sided pleural effusion was observed in two patients (4,8%) and was drained by pleural tube insertion during the hospitalization period. One patient (2,4%) developed cerebrovascular event characterized by left hemiparesia. All patients were discharged from the hospital without any complications.

### Follow-up period

All patients were called for clinical control by telephone and coronary arteriography was proposed. Three patients could not be reached. Two patients refused coronary arteriography. There were 3 late deaths; 2 were due to non-cardiac reasons (one patient died in a traffic accident and the other from pancreatic malignancy). The only cardiac death (2,4%) occured in the 34th postoperative month (sudden death). Thirty-five of 41 patients (85%) who accepted control coronary arteriography were in NYHA Class 1 functional capacity without recurrence of angina. Five patients described exertional dyspnea symptoms. One of them had already undergone percutaneous transluminal coronary angioplasty (PTCA) of the native proximal LAD due to the stenosis of the proximal LAD anastomosis. Another patient had undergone PTCA of the native right coronary artery due to the occlusion of the vein graft on this artery. One patient was in NYHA Class 3 functional capacity and was on anti-congestive medication against heart failure.

### Control coronary arteriographies

**Figure 1:**
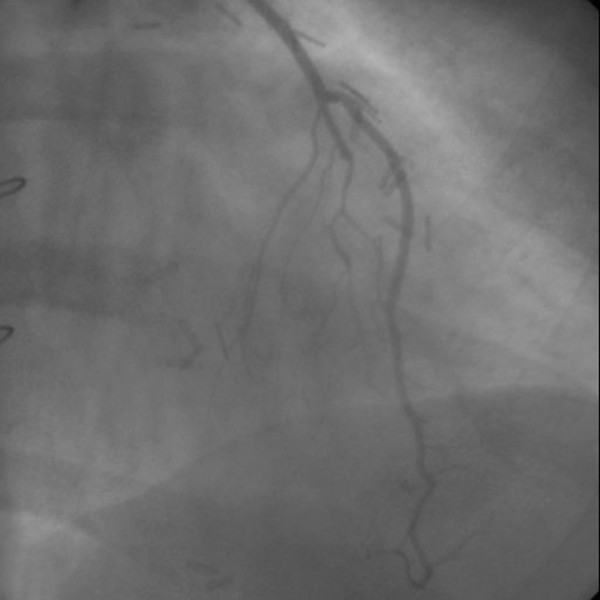
**Control arteriography of a sequential left internal thoracic artery to left anterior descending coronary artery anastomosis supplying the septal branches proximally and left ventricular apex distally**. Control arteriography of a sequential left internal thoracic artery to left anterior descending coronary artery anastomosis supplying the septal branches proximally and left ventricular apex distally.

**Figure 2:**
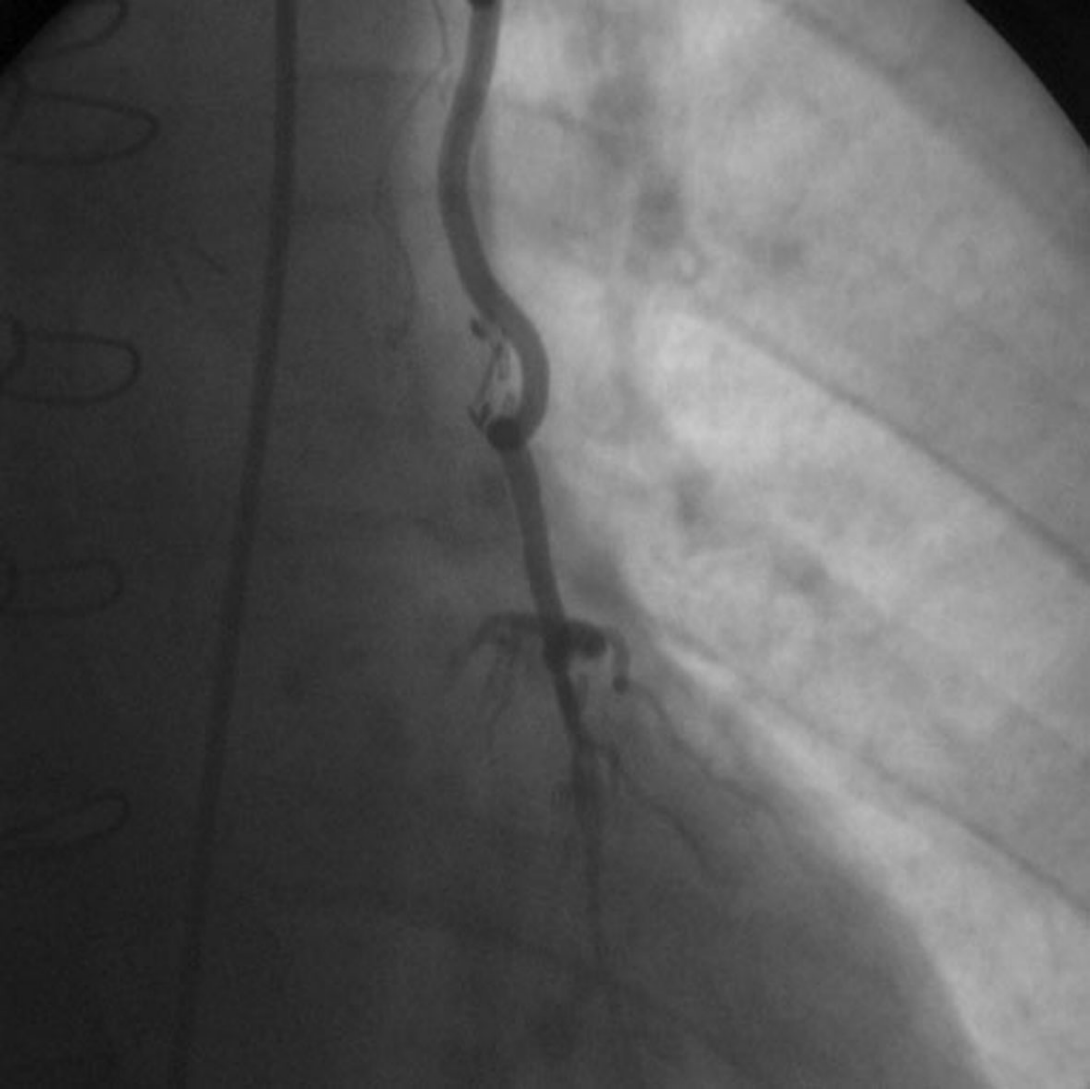
**Control arteriography of a sequential left internal thoracic artery to left anterior descending coronary artery anastomosis supplying the septal branches proximally and left ventricular apex distally**. Control arteriography of a sequential left internal thoracic artery to left anterior descending coronary artery anastomosis supplying the septal branches proximally and left ventricular apex distally.

Seventy five anastomoses were found to be fully open and patent (91,46%) among the 82 sequential LITA anastomoses (41 LITA grafts) on the LAD at a median follow-up period of 64 months (53 to 123 months). Of the 41 LITA grafts used for this purpose, 36 (87,8%) LITA grafts were found intact indicating a complete patency of the proximal and distal anastomoses (Figure 1, Figure 2).

Two LITA grafts (4 anastomoses) were totally occluded (4,87%). These patients were symptomatic and a re-operation is offered. In one patient, the proximal anastomosis of the LITA graft was 90% stenotic and this patient had already been treated with PTCA and stent implantation to the proximal LAD stenosis. In one patient, tight stenosis of the distal anastomosis line was observed (1,21%) while in another patient 70% narrowing in the LITA lumen after the proximal anastomosis was detected (1,21%). Medical treatment was decided for these two patients who had negative myocardial perfusion scanning studies with anti-anginal therapy.

## Discussion

The primary goal in coronary artery surgery should be the complete revascularization of all of the occluded or stenosed coronary arteries that supply viable myocardium with its best long-term results [3]. While the total number of coronary artery revascularization procedures decreases in the last years, the complexity and severity of each procedure increases in this surgery population. Cardiac surgeons are more and more confronted with patients suffering from diffusely and severly calcified coronary arteries [5]. In this patient population where the possibilities of conservative coronary artery surgery are limited, cardiac surgeons must add complementary revascularization techniques to their armementarium in order to offer these patients the benefits of complete coronary revascularization.

When the severely diseased coronary artery is the LAD, the revascularization of the septal branches as well as the apical part of the left ventricular myocardium gains importance. Several techniques have been proposed in the presence of an additional stenosis to the proximal LAD stenosis in order to revascularize as much possible as the anterior and apical parts of the left ventricular myocardium. Since Bailey's first coronary endarterectomy in the late 50's [6], the procedure has been the only weapon of the cardiac surgeons in these difficult cases for a long period. Despite the facts that higher rates of morbidity and mortality associated with the procedure [7,8], the coronary endarterectomy still keeps its place in these cases with improved results [5].

Extending the arteriotomy over the plaques on to the less diseased segments, so called long plaque-bridging arteriotomy, is another alternative technique proposed in diffuse LAD disease. Despite the good results reported with this technique [9], we assume that the graft patency might be impaired due to vascular wall pathology at the anastomosis site. Similar to this technique, long plaque-bridging arteriotomy of the LAD with additional vein patch reconstruction before the anastomosis is also an available technique in the presence of severe disease [4]. In the last two decades, the excellent results of LITA-LAD anastomosis, have made this graft the golden standard for LAD revascularization. With encouraging results of the LITA patency, a tendency to extend internal thoracic artery usage with bilateral or sequential internal thoracic artery techiques has become more and more popular in recent years [10,11]. With the pioneering efforts and excellent results of Tector [12], sequential LITA grafting gained popularity in coronary artery surgery and has become a very strong alternative in the presence of diffuse LAD disease.

At our department, sequential LITA anastomosis for severe LAD disease was advocated as the treatment of choice since late 90's. Over one hundred patients have undergone this procedure until today. Our goal with this technique is to revascularize septal branches of the LAD as well as the apical part of the left ventricular myocardium. In this particular group of patients with severely diseased LAD, we primarily check whether the diagonal artery to LAD connection is intact. In cases where this connection is intact, simple revascularization of the diagonal artery is usually effective to provide sufficient retrograde blood flow to the septal arteries and the distal stenosis of LAD is bypassed with another conduit. However, in cases where this connection is also stenosed, the LAD is first opened distal to the proximal stenosis and the severity of distal stenosis is judged through this opening. If a 1 mm coronary artery probe can not be advanced through this stenosis, the decision is made for sequential LITA revascularization. Mid LITA arteriotomy is performed and LITA to proximal LAD anastomosis is achieved in side to side fashion. Before the construction of the distal anastomosis, judgement of the flow from the distal end of the LITA and some bleeding from the distal coronary arteriotomy is critical to decide for the patency of the proximal anastomosis. In these patients, when the decision is sequential LITA grafting, we routinely begin intravenous nitroglycerine and diltiazem infusions and continue for two days, then the patient is followed with diltiazem for three months to attenuate LITA vasospasm risk.

As in our group, many other authors have also suggested that sequential LITA anastomoses as the best method to revascularize the LAD system which is diseased at multiple segments[1,13]). Although, endarterectomy is another option in such cases, we also believe that sequential LITA grafting to be a less invasive, safe and a more effective procedure in every possible patients, when compared to endarterectomy with its morbidity and mortality rates reaching significant differences in some reports especially when performed on the LAD [4,7,14].

The results of our study are also unique in being one of the largest series and providing the longest follow-up data in the litterature on this topic. The data and results obtained from the study are in accordance with other sequential LITA bypass studies [1,2,15] and are promising to research the behavior of sequential LITA only on the LAD. The results of sequential LITA to LAD anastomosis are similar to that of single LITA to LAD anastomosis (91,48% at a median follow-up period of 64 months) or even better and we did not observe a significant patency difference between the proximal and distal anastomoses. Additionally, we did not encounter any LITA hypoperfusion problem due to sequential use and we believe that the large coronary reserve in LITA sequential grafts may contribute to an improved long-term patency [16].

In the literature it has been shown that sequential bypass grafting has some advantages over the classical single bypasses. These are decreased impedance mismatch, decreased resistance to graft flow, and economical usage of the valulable grafts [2,17]. It is well documented that sequential grafting yields higher patency rates, especially when it is performed to small caliber and/or poor quality coronary arteries with poor run off [2,17,18]. Evidence may suggest that, distribution of inflow to multiple distal run offs may aid patency of the conduit especially when it is anastomosed to a poor target.

In conclusion, we strongly beleive that sequential LITA grafting of LAD is a safe alternative in the presence of severe LAD disease to achieve a complete revascularization of the anterior myocardium with patency rates not much differing from conventional single LITA to LAD anastomosis.

## Competing interests

The authors declare that they have no competing interests.

## Authors' contributions

MM, GC, CEY, AO act in data collection. MM, GC, CEY, MU, IMC, AO act in data interpretation and manuscript writing. MM, GC, MU, AA, CB act in study design and ciritical revision of the manuscript. All authors approved the final manuscript.
